# Chicago Medical Society

**Published:** 1884-04

**Authors:** 


CHICAGO MEDICAL SOCIETY.

The Chicago Medical Society held a meeting on the evening of February 4, 1884. After the usual routine of business had been transacted:

Dr. S. V. Clevenger gave a report of a case of "Paretic Dementia" in a female, whose peculiarities justify a brief synopsis. Kate M., æt. 37, Irish ; married ; was admitted to the Cook County Insane Asylum, April 12, 1883. She had been very intemperate before the insanity was noticed by friends. October, 1881, furor appeared and lasted three weeks. She remained in St. Joseph's Hospital of this city till a remission occurred, lasting till December 25, 1881, when, as her family state, she had "cramps," which lasted a week. She was then sent to Detroit (Michigan State Retreat), where she remained until the latter part of July, 1882, and little was elicited concerning her last remission, except that she was errabund. The first remission, her friends claim, was complete. Just prior to admission in the Cook County Asylum, delusions of persecution were observed. She exhibited marked paretic symptoms, tremulous oral and lingual muscles from the day of admission. Speech drawling, tremulous ; felt extremely well satisfied ; had unsystematized delusions of grandeur. She gave little or no trouble till August 30, 1883, when hemiplegia followed a convulsion, and next day she died without having been roused from stupor. It was ascertained that her father had died of "consumption of the bowels," and that her mother at the age of 60 died paralyzed ; also that this patient had never borne children, nor had she ever menstruated. An hereditary predisposition was denied. Necroscopy eight hours after death ; uterus very small, resembling that of a child of 16; left ovary atrophied ; os tincæ imperforate ; vagina patulous and large. A third nipple, well developed, below the left breast. The brain weighed 39 ounces after preparation in solutions for microscopical examination ; hence weights as here given have a relative value only. (The author is skeptical as to any importance attaching to absolute brain weights, and of late years will often omit weighing until the tissue is hardened.) Cerebellum weighed $4\frac{3}{4}$ ounces ; each hemisphere weighed within a grain of $16\frac{3}{8}$ ounces. The left side then can be considered under weight. Cholesterin abundant, especially in occipito-basilar regions ; right antero-posterior diameter of medulla slightly less than left. Isthmus weighed an ounce ; vermis of cerebellum warped toward left side, causing left aspect of the organ to appear larger than that of the right. Cortex did not pull off with pia mater loose from medullary substance, as noted in some cases by Spitzka. But in many parts of parietal and occipital regions the pial adhesions were sufficient to bring away the outer layers of cortex, imparting the ragged ulcerated appearance to which Rindfleisch called attention. Heterotopia found in anterior parietal region. Cortical pial adhesions occurred in the lowest extremity of the right occipital lobe, with connective tissue proliferations filling widened sulci.

This condition was extreme at base of first frontal convolutions on both sides, fibrous trabeculæ clubbed and twisted, extended downward from pia mater, covering four to five square inches, filling interstices left by shrunken gyri and atrophied gray substance. In the white substance, external to the right lobulous cuneus, a cone-shaped area of yellow softening with apex curved forward was found, measuring one inch in length, tapering from three lines in diameter. It was apparently the colloidal necrobiosis of a thrombosed terminal branch of the posterior cerebral artery. The gelatinous contents of the cone were mixed with detritus of the degenerated blood-vessel. Sections microscopically examined, afforded views of kinked and distorted vascular channels often twisted glomerularly. Knobbed vessels were frequent and perivascular spaces more so; some clear, others dotted with granular masses. Evidences of capillary stasis, general and decided, while dark bodies resembling embryo connective tissue corpuscles abounded. No well defined Meynert "spider-shaped" cells were discovered. The glanglionic elements were shrunken and their processes illy defined, a few large nerve cells with granule contents were observed lying in clear spaces, as though contracted from areas they once filled. Sclerosed patches abounded in the sections examined. With the exception of the lesions in the bregmatic region the post-Rolandiac parts were mainly involved. The ventricular endyma was nodulated in parts affording Spitzka's "ground glass" appearance. Comparatively few healthy ganglionic bodies were discerned in over a hundred sections taken from different parts of the cerebrum.

*Remarks.*—The frequency of paretic dementia occurring among women as compared with its occurrence among men. We have investigated thirty-nine different authorities, and have calculated the average proportion to be about one to eight. This includes Kiernan's cases. Neumann,* from not having seen a single case, denied the existence of paretic dementia among females. Sankey† inclines to the belief that sexual excess plays a part in the production of the disease, but the cases he cited show that there were, in addition to the sexual excesses, great vicissitudes.

Dickson ‡ favors the views offered by some writers, viz.: the liability, etc., but considers emotional mental strain as the great cause.

Luys || says that in existing social conditions males lead a much more active life than females, and are more subject to mental and emotional overstrain than females. Dr. Clevenger thinks the emotions preponderate in women, and even though carefully housed and cared for, nearly every woman has to bear at certain periods considerable tension of feeling, more probably in proportion to her possession of other mental qualities, which confer endurance.

Some authors state, the affection among the rural classes is markedly less frequent in females than is the case among the civic population, and is much more frequent with the hand-to-mouth, the trading and speculative classes, where the wife (Kiernan) takes an active part in her husband's business, than among the classes where she is confined at home. There is a marked relationship between the menopause and paretic dementia, as some writers have stated—there is a mental disturbance at this period predisposing to the psychosis. The prevalent type is the quiet form, which may account for the infrequency of its observation, and it is rarely met with among the female Irish in the South of Ireland.

In the discussion Dr. J. G. Kiernan stated, that even admitting females to be more emotional than males, when they are confined to their household duties, which are more monotonous than those of the male class of a speculative nature, the per-

* Lehrbuch der Psychiatrie, 1859.

† Lectures on Mental Disease.

‡ Medicine in Relation to Mind.

|| Maladies Mentales.

centage of cases among them is one to every 6 or 8 of males. In France there is a middle class (the Bourgeoise) where a wife takes an active part in her husband's business, and paretic dementia occurs in them. Regarding the ætiology, so far as alcoholic excesses go, statistics are worthless. In 160 cases reported, 116 were intemperate, but this is a symptom of the affection, instead of a cause. In British Guiana, where the people indulge in excesses of a sexual nature as well as the alcoholic, and also where syphilis prevails to a considerable extent, the disease is unknown, although at Berbes, where the asylum is located in British Guiana, the Superintendent reported one case, that of a male, who came there from Europe, and was afflicted with the malady ere he came to Berbes.

Some French authors state syphilis is not a predisposing cause, whilst Dr. Clevenger thinks paresis is very largely due to inherited or acquired syphilis.

Dr. R. Tilley thought if emotional strain had anything to do as a cause of this debility, that a Board of Trade speculator was much more apt to have it than a mechanic who gets his pay regularly, and yet in the large portion constituting the business class we scarcely ever hear of cases.

Dr. Kiernan stated he had no doubt but that the theory regarding emotional strain is true. The easy-going class in Ireland (Celts) do not have it, as may be illustrated by their not worrying as to how to prosper. In this city the emotional strain in those living from hand to mouth are the largest proportion who have this trouble; while Jessen, a Scandinavian alienist, traced all cases to congenital or acquired syphilis.

This topic being concluded, Dr. Henry J. Reynolds read an interesting paper on the "Treatment of Urethral Stricture."

Dr. W. L. Axford was much interested in the remarks embodied in the paper just read, and said the author took the extreme views advanced by Dr. Otis, of New York, and that the advances in urethral surgery to-day are due principally to Otis. In strictures of large caliber the best way to treat them is to cut them, as there is but little risk in doing so. In some cases of stricture, of traumatic origin, this method is hardly admissible, and Syme's operation is then preferable—external perineal urethrotomy. The value of the acorn sound is greater in determining a stricture than that of the urethrometer, but to use the acorn instrument, the meatus may have to be incised. I want to ask the writer if he thinks in all cases of gleet there is stricture? Answered, yes. Then in all cases of stricture is there gleet? Answered, no.

Dr. C. E. Webster cited briefly a number of cases of stricture of large caliber he had seen operated upon. The first patient was in the first stage of pneumonia, and subsequently died of lung trouble. The second patient had organic disease of the heart, and died of the heart affection. A third case operated on had Potts' Disease. None of these patients were benefited by the operation. The same surgeon, who is a teacher in a well known school, operated for phymosis in a case of bow legs, expecting thereby to relieve this deformity, as no other treatment for the bow legs was recommended. Then the following two questions were asked the reader of the paper: First, whether he would operate upon a stricture of large caliber if no local symptoms were present; and second, whether acorn sounds of large size, which pass by dilating the urethra, may not indicate a stricture, where the condition is not pathological, but simply due to a varying degree of elasticity along the tract of a healthy organ?

Dr. C. T. Fenn gave his experience as a physician, (more than that of a surgeon) in introducing the finest sound, by reciting a case that not long since had been under his care. One of our well-known surgeons also saw the case with him in consultation; but in essaying to pass the smallest steel sound had failed, and then resorted to aspiration of the bladder over the pubes. He had previously, however, introduced a filiform bougie, and the passage was effected by "passing it around" the stricture, as was determined at the autopsy, when the stricture was dissected and found to be "fibrous," and in connection with it there was infiltration of urine, which caused death. The speaker did not agree with the author of the paper in wanting to operate on so many of this class of cases, and thought the operation should be approched with no little caution, as the dark urethral channel could not be seen whilst operating upon it.

Dr. R. Tilley asked the reader why he thought it was necessary in his cases to render the urine alkaline before operating? and why this is better than the normal urine, which is but slightly acid? Normal urine. he believed, was practically innocuous for wounds.

Dr. G. C. Paoli's experience and observation warranted his saying that only temporary benefit was derived from cutting operations, and that we should be very careful in making these incisions. Exudation takes place in two or three years at longest. He has treated between five and six hundred cases of stricture of the urethra by gradual dilatation. His patients were satisfied with this continual method, and all were successful, so far as he knew, without the cutting. He believed one should attain the object by the method of gradual dilatation, and that it is far preferable. He then gave a history of a case which was operated on 34 years ago in Cincinnati. There was considerable hæmorrhage at the time; temporary success followed, but in a short time the caliber of the canal become lessened and indurated. Every fortnight for a long period of time he passed a No. 8 or 9 Otis curve sound.

Dr. L. H. Montgomery thought stricture in the male a cause of sterility, and that the power of propagation was in many instances lessened by it, and inquired as to this; also of the writer, how long he required a patient to take an alkali, before the urine became alkaline, and if bicarbonate of soda in ʒss doses was not superior to the citrate of potash in rendering urine alkaline?

Dr. D. W. Graham said, that one of our eminent men in this city stated long ago, he thought a previous attack of gonorrhœa would cause sterility in the male. To cut a stricture is malpractice, and to cut what is revealed by an acorn sound, he regarded also to be malpractice.

Dr. Reynolds in closing said, he thought it was essentially necessary to dilute the urine, to do so, a patient will not void it so frequently, and in cystic irritation it should be rendered alkaline. In answer to Dr. Webster, he thought the condition to which he alluded would be extremely rare, a patient would not know he had stricture, unless local symptoms presented. For then a patient will consult me, and I probably should then advise an operation. Regarding one speaker's interrogatory about stricture being a cause of sterility, he thought this was not the case; also that the bicarbonate of soda or potash, given for two or three days, was equally good with the citrate of potash to produce alkalinity of the urine.

The resignation of Dr. D. F. McPherson was accepted on account of his removal to Buffalo, N. Y.

Adjourned. L. H. M.

---

CHICAGO MEDICAL SOCIETY.

